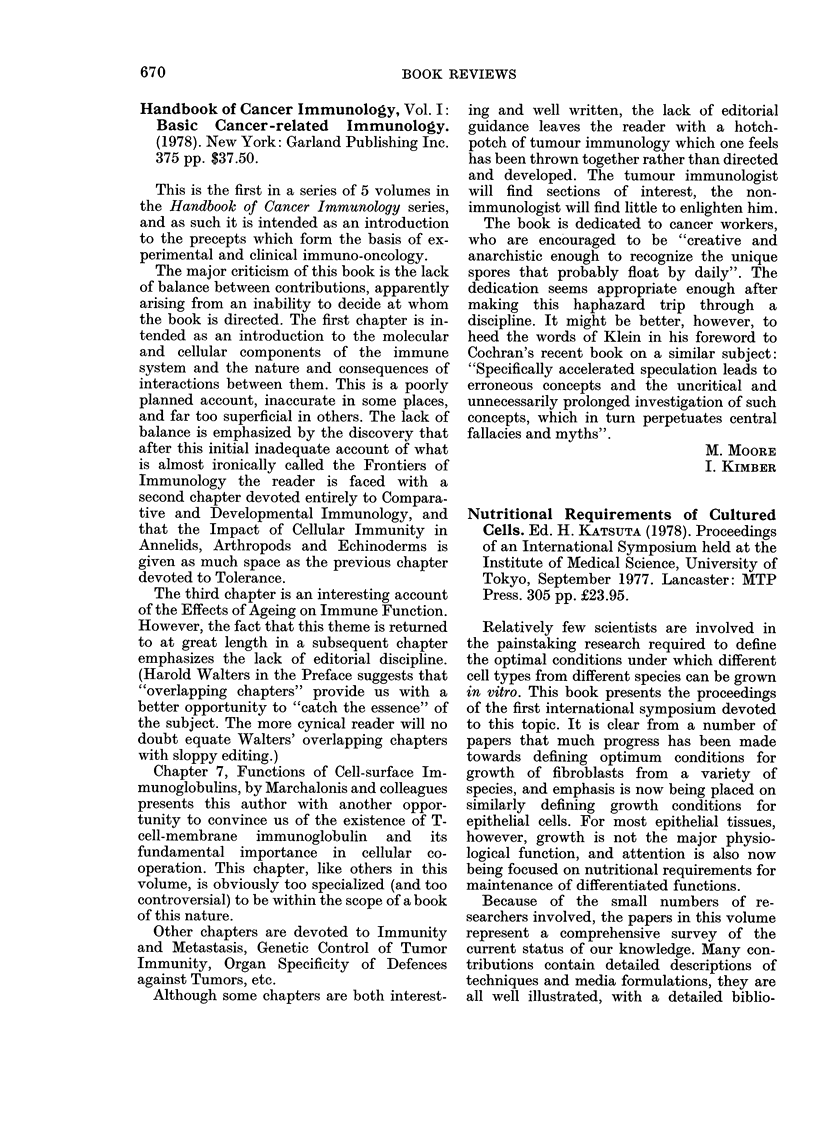# Handbook of Cancer Immunology, Vol. I: Basic Cancer-related Immunology

**Published:** 1979-10

**Authors:** M. Moore, I. Kimber


**Handbook of Cancer Immunology,** Vol. I: **Basic Cancer-related Immunology.** (1978). New York: Garland Publishing Inc. 375 pp. $37.50.

This is the first in a series of 5 volumes in the *Handbook of Cancer Immunology* series, and as such it is intended as an introduction to the precepts which form the basis of experimental and clinical immuno-oncology.

The major criticism of this book is the lack of balance between contributions, apparently arising from an inability to decide at whom the book is directed. The first chapter is intended as an introduction to the molecular and cellular components of the immune system and the nature and consequences of interactions between them. This is a poorly planned account, inaccurate in some places, and far too superficial in others. The lack of balance is emphasized by the discovery that after this initial inadequate account of what is almost ironically called the Frontiers of Immunology the reader is faced with a second chapter devoted entirely to Comparative and Developmental Immunology, and that the Impact of Cellular Immunity in Annelids, Arthropods and Echinoderms is given as much space as the previous chapter devoted to Tolerance.

The third chapter is an interesting account of the Effects of Ageing on Immune Function. However, the fact that this theme is returned to at great length in a subsequent chapter emphasizes the lack of editorial discipline. (Harold Walters in the Preface suggests that "overlapping chapters" provide us with a better opportunity to "catch the essence" of the subject. The more cynical reader will no doubt equate Walters' overlapping chapters with sloppy editing.)

Chapter 7, Functions of Cell-surface Immunoglobulins, by Marchalonis and colleagues presents this author with another opportunity to convince us of the existence of T-cell-membrane immunoglobulin and its fundamental importance in cellular cooperation. This chapter, like others in this volume, is obviously too specialized (and too controversial) to be within the scope of a book of this nature.

Other chapters are devoted to Immunity and Metastasis, Genetic Control of Tumor Immunity, Organ Specificity of Defences against Tumors, etc.

Although some chapters are both interesting and well written, the lack of editorial guidance leaves the reader with a hotchpotch of tumour immunology which one feels has been thrown together rather than directed and developed. The tumour immunologist will find sections of interest, the non-immunologist will find little to enlighten him.

The book is dedicated to cancer workers, who are encouraged to be "creative and anarchistic enough to recognize the unique spores that probably float by daily". The dedication seems appropriate enough after making this haphazard trip through a discipline. It might be better, however, to heed the words of Klein in his foreword to Cochran's recent book on a similar subject: "Specifically accelerated speculation leads to erroneous concepts and the uncritical and unnecessarily prolonged investigation of such concepts, which in turn perpetuates central fallacies and myths".

M. Moore
I. Kimber